# Terlipressin and albumin for type 1 hepatorenal syndrome: does bacterial infection affect the response?

**DOI:** 10.1186/s40064-015-1625-z

**Published:** 2015-12-23

**Authors:** Reskan Altun, Murat Korkmaz, Emre Yıldırım, Serkan Öcal, Enver Akbaş, Haldun Selçuk

**Affiliations:** Department of Gastroenterology, Baskent University, Ankara, Turkey

**Keywords:** Hepatorenal syndrome, Terlipressin, Albumin, Active bacterial infection

## Abstract

Vasoconstrictor therapy with terlipressin and concomitant albumin can improve renal function in patients with hepatorenal syndrome (HRS) type 1, but the efficacy of therapy in patients with active infection is controversial. The aim of this study was to investigate the efficacy, adverse effects, and predictors of terlipressin therapy and to find out whether there was a difference in response rates between the patients with or without active infections. Data of 58 patients with type 1 HRS treated with terlipressin and albumin were retrospectively evaluated. Twenty-six patients (44.8 %) showed complete response to treatment. Response rates of patients with or without active bacterial infection were 47 and 43.9 %, respectively (*p* > 0.05). Only baseline serum creatinine level was significantly related to response in univariate/multivariate analyses (*p* < 0.05). Twenty-three patients (39.6 %) developed adverse effects probably related to treatment. In 8.6 % of patients, treatment was discontinued because of adverse effects of therapy. Four patients (6.9 %) developed ischemic adverse events, including nonfatal myocardial infarction, intestinal ischemia, and cutaneous necrosis. Terlipressin plus albumin therapy improved renal function in nearly half of patients with type 1 HRS. Thus, it seems a reasonable treatment for patients with active bacterial infections. Baseline serum creatinine level is a potential predictor of terlipressin response.

## Background

Hepatorenal syndrome (HRS) is a potentially reversible, functional renal failure that occurs in patients with advanced liver disease. HRS is generally characterized by increased serum creatinine, azotemia, reduced diuresis, increased urine osmolarity, and reduced urine sodium values without signs of organic kidney damage (Salerno et al. 2007).

Vasoconstrictor therapy with terlipressin (a vasopressin-V1 receptor agonist), and concomitant albumin can improve renal function in patients with type 1 HRS (Ortega et al. 2002; Solanki et al. 2003; Martín-Llahi et al. 2008; Sanyal et al. 2008; Neri et al. 2008). Terlipressin seems to reverse the systemic and splanchnic vasodilatation, increase blood pressure, and decrease the hepatic-renal arterial resistance in cirrhotic patients with and without ascites (Narahara et al. 2009).

Many studies, including several randomized controlled trials, have shown that terlipressin is associated with a low incidence of adverse effects (Ortega et al. 2002; Solanki et al. 2003; Martín-Llahi et al. 2008; Sanyal et al. 2008; Neri et al. 2008; Moreau et al. 2002; Mulkay et al. 2001; Halimi et al. 2002). In the recent guidelines, a starting terlipressin dose of 0.5–1.0 mg/4–6 h by IV bolus has been recommended (Salerno et al. 2007). However, many unanswered questions remain regarding the optimal dose, optimal route of administration, and the potential adverse effects, including severe ischemic events.

According to the revised diagnostic criteria reported in 2007, patients with renal failure and active bacterial infections, without septic shock, are also considered as having HRS. Additionally, in the recent guidelines, starting the treatment of HRS before a complete recovery from the infection has been recommended (Salerno et al. 2007).

In this article, we have investigated a cohort of subjects with liver cirrhosis who fulfill the criteria of type 1 HRS according to the guidelines used at the time of diagnosis. The primary aim of this study was to investigate the efficacy, possible adverse effects, and potential predictors of terlipressin therapy. The secondary aim was to find out whether there was a difference in terms of complete response to treatment with terlipressin between the patients with or without active infections.

## Methods

Data of 58 patients meeting the criteria of type 1 HRS, as proposed by the International Ascites Club at the time of diagnosis (Salerno et al. 2007; Arroyo et al. 1996), who underwent treatment with terlipressin at our hospital, were retrospectively reviewed. The protocol of this study was in accordance with ethical standards of Baskent University research committee and patients provided written informed consent to participate in the study.

All patients with suspected HRS were evaluated with the protocol that includes diuretic withdrawal, assessment of other possible causes of prerenal azotemia, and a trial of plasma expansion with albumin. To rule out renal parenchymal diseases, urine analyses and abdomen ultrasonography were performed. The presence of infection was evaluated with blood, sputum, urine, and ascitic fluid cultures as well as serum, and ascitic fluid leukocyte counts, serum C-reactive protein levels, and chest radiographs. In all patients, cultures were routinely obtained before the start of antibiotic therapy. Patients who fulfilled the criteria of type 1 HRS according to the guidelines used at the time of diagnosis were treated (Salerno et al. 2007; Arroyo et al. 1996). Thirty of 58 patients were diagnosed and managed based on previous criteria since the new criteria had yet to be published at that time. In 17 of the 28 patients diagnosed with the new criteria, an active bacterial infection was present. Ten of 17 patients had spontaneous bacterial peritonitis (presence of a polymorphonuclear leucocyte count >250/mm^3^ in ascitic fluid, with or without positive culture). Other infections were as follows: three patients with bacterial pneumonia, three patients with urinary tract infection, and one patient with spontaneous bacteremia (presence of positive blood cultures without evident source of infection).

The exclusion criteria were as follows: presence of severe extrahepatic condition, including cardiovascular (coronary and/or peripheral arterial disease) and neurological diseases, septic shock, and hepatocellular carcinoma outside the Milan criteria.

All patients were hospitalized in the intensive care unit before therapy. Before therapy with terlipressin, all patients were monitored noninvasively. Arterial pressure and urinary output were checked every 4 h. Cardiac rhythm was monitored continuously. An electrocardiogram was performed before and during treatment when needed. Patients with cardiac symptoms, and typical signs of ischemia on the first electrocardiogram were not included the study. All patients had an echocardiographic study within the 6 months period before treatment. Physical examination and routine laboratory tests were performed in all patients before the initiation of therapy and daily intervals during treatment.

During the first 3 days of treatment, terlipressin (glypressin 1 mg; Ferring GmbH, Kiel, Germany) was administered at a dose of 0.5–1 mg every 4 h as an intravenous bolus in 50 patients and as a short-period infusion (15–30 min) in eight patients. If after the first 3 days, serum creatinine decreased at least 25 % of the pretreatment values, the dose remained unchanged. In patients whose serum creatinine did not decrease at least 25 % of the pretreatment values within the first 3 days, the dose was increased up to a maximum of 2 mg/4 h. Terlipressin was given until serum creatinine decreased below 1.5 mg/dl and urine output increased above 500 ml/day. We did not use a fixed maximum time period for terlipressin treatment. Patients received 40 g of albumin during the first 24 h, followed by 20 g/day. Patients with proven bacterial infection were treated with wide-spectrum antibiotics including second- and third-generation cephalosporins and carbapenems according to antimicrobial susceptibility. Prophylactic antibiotic therapy was not given during the treatment.

Severity of liver disease was assessed by the Child–Pugh classification. Patients were followed until death, discharge, or liver transplantation. Patients developing recurrence were not evaluated again. Adverse effects of therapy were also recorded.

### Statistical analysis

Results are presented as mean ± standard deviation (SD) and median (minimum–maximum). Continuous variables were compared with Student’s *t* test, and Mann–Whitney test. Categorical variables were compared with Pearson Chi square and Fisher’s exact tests. Univariate and multivariate logistic regression analyses were performed to identify predictors of response. A *p* < 0.05 was considered as statistically significant. All analyses were done using the Statistical Package for the Social Sciences (SPSS) 19.0 (SPSS Inc. Chicago, IL, USA).

## Results

Data of 58 patients with type 1 (HRS), who underwent treatment with terlipressin and albumin, were evaluated. Among 58 patients, 77.6 % (*n* = 45) and 22.4 % (*n* = 13) were men and women, respectively. Patients had a mean age of 52.6 ± 10.6 years. Cirrhosis was caused by viral hepatitis (28 patients with hepatitis B, 6 patients with delta hepatitis, and 2 patients with hepatitis C) in 62.1 % (*n* = 36) of patients. Severity of cirrhosis defined by Child–Pugh score was Class A in 6.9 % (*n* = 4), Class B in 27.6 % (*n* = 16), and Class C in 65.5 % (*n* = 38). Ascites was present in 87.9 % (*n* = 51) of patients. Demographic and clinical characteristics of the patients before therapy are shown in Table 1.

**Table 1:** Demographic and clinical characteristics of the 58 patients

Age^a^ (years)	52.6 ± 10.6
Gender (M/F)	45/13
Child–Pugh class (A/B/C)	4/16/38
Etiology (viral hepatitis/others)	36/22
Ascites (±)	51/7
Active bacterial infection (±)	17/41
Serum total bilirubin^a^ (mg/dl)	2.8 ± 0.8
Serum albumin^a^ (g/dl)	2.7 ± 0.6
Serum creatinine^a^ (mg/dl)	2.2 ± 0.8
Serum Na^a^ (mEq/L)	129 ± 5
Urine volume^a ^(ml/day)	339 ± 190
MAP^a^ (mmHg)	78 ± 9

*MAP* mean arterial pressure

^a^Mean ± SD

Twenty-six patients (44.8 %) showed response to the treatment. Demographic and clinical characteristics of responders and nonresponders are summarized in Table 2. Of note, in 11 of 27 patients who did not show response to the treatment, the 24 h urine volume was increased over 500 ml/day during treatment. Baseline serum creatinine was significantly low, and 24 h urine volume was significantly high in responders compared with nonresponders (*p* < 0.05).

**Table 2:** Demographic and clinical characteristics of responders and nonresponders before therapy

	Responders (*n* = 26)	Nonresponders (*n* = 27)	*p*
Age^a^(years)	53.9 ± 7.7	53 ± 11.4	NS
Gender (M/F)	19/7	24/3	NS
Etiology of cirrhosis (viral hepatitis/others)	18/8	16/11	NS
Child–Pugh class (A/B/C)	3/7/16	1/7/19	NS
Child–Pugh score	9.8 ± 2.5	10.2 ± 2	NS
Ascites (±)	22/4	25/2	NS
Active bacterial infection (±)	8/18	7/20	NS
Serum total bilirubin^a^ (mg/dl)	2.6 ± 0.8	2.9 ± 0.8	NS
Serum albumin^a^ (g/dl)	2.6 ± 0.6	2.7 ± 0.5	NS
Serum creatinine^a^ (mg/dl)	1.9 ± 0.4	2.5 ± 1	<0.05
Serum Na^a^ (mEq/L)	129 ± 6	128 ± 4	NS
Urine volume^a ^(ml/day)	400 ± 186	285 ± 181	<0.05
MAP^a ^(mmHg)	80 ± 10	77 ± 9	NS

*NS* nonsignificant,* MAP* mean arterial pressure

^a^Mean ± SD

Seventeen of the 58 patients (29.3 %) had an active bacterial infection, without septic shock. In the group of patients with HRS and active bacterial infection, 8 of 17 patients (47 %) showed response treatment with terlipressin and albumin, while 18 of 41 patients (43.9 %) without active infection showed response (*p* > 0.05).

The variables, age, gender, etiology of cirrhosis, Child–Pugh scores, existence of ascites, existence of active bacterial infection, serum total bilirubin, serum albumin, serum creatinine, serum sodium levels, 24 h urine volume, and mean arterial pressure (MAP) were evaluated as potential predictors of terlipressin and albumin response in both univariate and multivariate analyses (Table 3). Only baseline serum creatinine level (<2.5 mg/dl) was significantly related to response (*p* < 0.05).

**Table 3:** Univariate analysis for potential predictors of terlipressin and concomitant albumin response

Variables	OR (95 % CI)	*p*
Age		
>50	2.0 (0.67–5.98)	0.21
≤50		
Gender		
Male	2.9 (0.67–12.9)	0.15
Female		
Etiology of cirrhosis		
Viral hepatitis	1.5 (0.49–4.80)	0.45
Others		
Child–Pugh score		
≤10	1.7 (0.57–5.05)	0.33
>10		
Ascites		
Yes	2.2 (0.37–13.63)	0.36
No		
Active bacterial infection		
Yes	1.2 (0.38–4.20)	0.69
No		
Serum total bilirubin (mg/dl)		
≤3	2.0 (0.67–5.98)	0.21
>3		
Serum albumin (g/dl)		
≥2.8	1.51 (0.49–4.57)	0.46
<2.8		
Serum creatinine (mg/dl)		
<2.5	6.13 (1.47–25.44)	0.01
≥2.5		
Serum Na (mEq/L)		
≥130	2.45 (0.76–7.88)	0.13
<130		
Urine volume (ml/day)		
≥400	2.33 (0.76–7.08)	0.13
<400		
MAP^a^ (mmHg)		
≥80	2.31 (0.77–6.98)	0.13
<80		

*CI* confidence interval, *OR* odds ratio

^a^ *MAP* mean arterial pressure

Twenty-three patients (39.6 %) developed adverse effects probably related to the treatment, and in 5 of 58 patients (8.6 %), treatment was discontinued because of treatment-emergent adverse effects. Sixteen patients (27.6 %) developed diarrhea, which subsided after cessation (1 patient), lowering dose (3 patients), or changing administration protocol to short-period infusion (12 patients) of terlipressin therapy. Three patients (5.2 %) developed abdominal pain, which subsided after changing administration protocol to short-period infusion (one patient) or lowering dose (two patients) of terlipressin therapy. Four patients (6.9 %) developed ischemic adverse events (myocardial infarction, intestinal ischemia, cutaneous necrosis). In one patient with cutaneous necrosis and two patients with intestinal ischemia, symptoms disappeared after terlipressin withdrawal. Finally, one patient developed nonfatal myocardial infarction during terlipressin therapy.

HRS recurred in 12 (46.1 %) of 26 patients who responded to treatment. Mean survival time was significantly greater in responders compared with nonresponders (42 ± 6.2 and 21.8 ± 4.3 days, respectively, *p* < 0.05). Five patients had undergone transplantation. The transplant-free survival at 15 and 30 days of treatment was 54.7 and 32 %, respectively.

## Discussion

Splanchnic vasodilation in cirrhotic patients may reduce effective arterial filling, resulting in renal vasoconstruction (Moller and Henriksen 2004). Intravascular volume expansion with vasoconstrictor drugs may reverse this vicious cycle and contribute to the treatment of patients with HRS or at risk of HRS (Krag et al. 2007). The achievement of optimal volume expansion is difficult in these patients with standard static hemodynamic measurements (Marik et al. 2008). Volume overload should be avoided owing to the risk of expanding third spaces and the subsequent development of abdominal compartment syndrome, which could reduce renal perfusion (Dalfino et al. 2008). Studies have shown that the use of vasocontrictors, such as noradrenaline and terlipressin in conjunction with albumin, improves renal function in patients with HRS compared with the use of vasoconstrictors or albumin alone (Ortega et al. 2002; Solanki et al. 2003; Martín-Llahi et al. 2008; Sanyal et al. 2008; Neri et al. 2008; Sharma et al. 2008). Randomized controlled studies have shown that the HRS reversal rates achieved with terlipressin and albumin treatment ranged between 34 and 81 % (Sanyal et al. 2008; Neri et al. 2008). The reason of this wide spectrum may be due to both complexity of the pathogenesis and difficulties in the assessment of effective treatment. In our study, similar to many randomized controlled trials (Solanki et al. 2003; Martín-Llahi et al. 2008), 44.8 % of patients showed response to treatment.

According to the 2007 consensus report, patients with renal failure and active bacterial infections, without septic shock, are also diagnosed as having HRS. Additionally, starting the treatment before a complete recovery from the infection has been recommended (Salerno et al. 2007). The results of the study published by Barreto et al. in 2014 has supported this recommendation. They showed that type 1 HRS associated with infections is not reversible in two-thirds of patients with treatment of infection only (Barreto et al. 2014). This result has pointed to the terlipressin and albumin treatment again. The gap in the knowledge of type 1 HRS associated with infection has filled with the study of Rodriguez et al. Their study provides evidence indicating that terlipressin and albumin is also effective for the treatment of type 1 HRS associated with sepsis (Rodriguez et al. 2014).

In our study, we also compared groups of patients with or without active infection and found that there is no statistically significant difference between HRS reversal rates (47 and 43.9 %, respectively, *p* > 0.05) as mentioned by Rodriguez et al. 2014. In many patients, type 1 HRS is triggered by bacterial infections given the aggravation of splanchnic vasodilatation by endotoxemia and cytokine overproduction (Arroyo et al. 2007). As shown in our study, it seems reasonable to treat these patients with terlipressin and albumin concomitantly with antibiotics.

Predictors of response to terlipressin and albumin therapy in patients with type 1 HRS in previous studies included baseline Child–Pugh and MELD scores, serum creatinine, serum bilirubin levels, 24 h urine volume, and the presence of an increase in MAP during therapy (Sanyal et al. 2008; Neri et al. 2008; Moreau et al. 2002; Boyer et al. 2011; Nazar et al. 2010). In our study, baseline serum creatinine was significantly low, and urine volume was significantly high in responders, whereas significant differences did not exist in terms of age, sex, serum bilirubin, serum albumin, serum sodium levels, and baseline MAP between responders and nonresponders. In a recent study (Boyer et al. 2011), it was reported that the most consistent predictor of response was baseline serum creatinine as observed in our study. Meanwhile, baseline serum creatinine was not associated with response in another study (Nazar et al. 2010). We think that predictors of response to terlipressin therapy are not well established in the literature, and further studies are needed.

Terlipressin has a low adverse effect profile comparing to other vasopressors that have been used for the treatment of HRS. However, it is recommended that terlipressin be used in an intensive care unit with close hemodynamic monitoring because of potentially serious ischemic adverse events (European association for the study of the liver 2010). In a randomized controlled trial including 112 patients with type 1 HRS, it was reported that serious adverse effects were mostly cardiovascular and were observed in 9 % of patients treated with terlipressin and albumin. For this reason, in that study, treatment was discontinued in 5.4 % of patients (Sanyal et al. 2008). In another randomized controlled trial, cardiovascular complications occurred in 22 % of patients, but terlipressin therapy was discontinued in only one patient because of high blood pressure (Martín-Llahi et al. 2008). By comparison, a meta-analysis including 10 clinical trials, published before these two abovementioned trials reported that the pooled rate of withdrawal, and adverse effects of terlipressin therapy were 0 and 29 %, respectively. In our study, 39.6 % of patients developed adverse effects related to treatment, and in 8.6 % of patients, treatment was discontinued. These adverse effects were diarrhea (27.6 %), abdominal pain (5.2 %), and ischemic adverse events (6.9 %), including nonfatal myocardial infarction, intestinal ischemia, and cutaneous necrosis. Other adverse effects reported before, such as cardiac arrhythmias, hypertension, respiratory failure, and bronchospasm, were not observed. Further, none of our patients died because of terlipressin therapy.

In our study, adverse effects related to terlipressin therapy occurred after a mean of 2.6 days. Thus, it should be emphasized that close monitoring for adverse effects is required during the first few days of treatment, in particular, when treating patients with comorbidities.

Some authors suggest that continuous terlipressin infusion may have less severe adverse effects with the same response rates compared with intermittent IV bolus administration (Gerbes et al. 2009). However, this hypothesis has not been proven in any randomized, controlled trials yet. In our study, terlipressin was administered initially as an IV bolus in 50 patients, and as short-period infusion (15–30 min) in eight patients. We did not compare the groups in terms of adverse effects because of the small number of patients. However, adverse effects subsided after changing the administration protocol from IV bolus to short-period infusion in 56.5 % of patients who developed adverse effects. Thus, it should be noted that short-period infusion may be an alternative of IV bolus administration if the onset of adverse events occurs.

Our study has some limitations. First, it is a retrospective study with a relatively small number of patients. Moreover, predictive factors of response should be analyzed in a prospective study.

In conclusion, our study results indicate that terlipressin plus albumin therapy improves renal function in nearly half of patients with type 1 HRS. Further, it is reasonable to treat patients with type 1 HRS and active bacterial infections because of similar response rates compared with patients without active infections. Nevertheless, clinicians should be vigilant for severe adverse effects. We think that future studies should be focused on predictors of response to terlipressin therapy and therapies for prophylaxis as well as new pharmacological agents for nonresponders.
